# Preimplantation Genetic Testing for Aneuploidy in Recurrent Pregnancy Loss: A Fertility Success Story

**DOI:** 10.7759/cureus.84084

**Published:** 2025-05-14

**Authors:** Apostolos C Ziogas, Polyxeni-Natalia Liasidi, Afroditi Katouna, Anastasia Koutsouni, Konstantina Faki, Evangelia Karangeli, Efthymia Thanasa, Anna Thanasa, Ioannis Thanasas, Emmanouil M Xydias

**Affiliations:** 1 School of Health Sciences, Faculty of Medicine, University of Thessaly, Larissa, GRC; 2 Outpatient Gynecology Clinic, OB/GYN SPOT, Larissa, GRC; 3 Department of Obstetrics and Gynecology, Agios Dimitrios General Hospital, Thessaloniki, GRC; 4 IVF Clinic, IASO Thessalias, Larissa, GRC; 5 Department of Histology, Aristotle University of Thessaloniki, Thessaloniki, GRC; 6 Department of Anatomy, Aristotle University of Thessaloniki, Thessaloniki, GRC; 7 Department of Obstetrics and Gynecology, General Hospital of Trikala, Trikala, GRC; 8 Department of Obstetrics and Gynecology, Aristotle University of Thessaloniki, Thessaloniki, GRC

**Keywords:** assisted reproduction technology, euploidy, infertility, karyotype, preimplantation genetic testing for aneuploidy (pgt-a), recurrent pregnancy loss

## Abstract

Recurrent pregnancy loss (RPL) is a challenging condition in the field of assisted reproduction, with various potential causes that may be maternal or fetal in origin. When fetal factors are involved, preimplantation genetic testing for aneuploidy (PGT-A) serves as an effective method to assess embryo genetic integrity, helping identify viable embryos with a higher likelihood of implantation and live birth. This report describes the case of a 30-year-old woman with a history of recurrent miscarriages. After a comprehensive evaluation of possible maternal and paternal factors, and three additional pregnancy losses confirmed to be due to aneuploidy, she underwent in vitro fertilization combined with PGT-A. This resulted in the identification of seven euploid blastocysts. A single embryo transfer led to an ongoing pregnancy, highlighting the clinical utility of PGT-A in the management of RPL.

## Introduction

Recurrent pregnancy loss (RPL) is defined as the occurrence of at least two histologically or ultrasonographically confirmed pregnancy losses from conception up to 24 weeks of gestation [1,2], although some investigators support defining it as three or more losses [3]. RPL is a multifactorial condition with potential causes stemming from both embryonic and maternal factors. Most experts recommend initiating a thorough diagnostic evaluation after the second miscarriage [4]. This evaluation typically includes a detailed medical history, focusing on the number of losses and the gestational age at which they occurred, along with a physical examination and a series of specialized laboratory and imaging tests [4,5].

After completing the diagnostic workup, treatment options, including in vitro fertilization (IVF) and other assisted reproductive technology (ART) techniques, may be considered based on the identified etiologic factors and the preferences of the individual or couple, with the goal of increasing the likelihood of achieving a successful full-term pregnancy.

While numerous maternal causes of RPL have been identified, such as thin endometrium, uterine fibroids, or tubal obstruction, several treatment options are available to address these issues and improve the chances of conception and live birth [6-8]. However, when RPL is attributed to embryonic factors, the only legally and ethically acceptable approach is to select high-quality embryos for transfer. Traditionally, embryo selection has relied on morphological criteria [9]. With recent advances in genomics, however, more accurate techniques are now available to assess embryo quality prior to implantation, primarily through preimplantation genetic testing (PGT) [10].

PGT involves analyzing embryonic genetic material, most commonly obtained through trophectoderm biopsy at the blastocyst stage, to enable the selection and transfer of genetically normal embryos, thereby improving implantation rates [10]. Several types of PGT exist, including testing for polygenic disorders (PGT-P), structural rearrangements (PGT-SR), and monogenic disorders (PGT-M). Among these, the most widely used is PGT for aneuploidy (PGT-A) [10]. PGT-A is designed to detect de novo aneuploidies, as well as subchromosomal deletions or duplications in embryos from karyotypically normal parents. By enabling the transfer of only euploid embryos, PGT-A reduces the risk of miscarriage and pregnancy-related complications, thereby improving the likelihood of a full-term pregnancy.

This report presents a case of a successful ongoing pregnancy achieved through the transfer of PGT-A-tested embryos in a couple with a history of RPL. It also discusses current literature-based recommendations on evaluating women with recurrent miscarriages and the role of PGT-A in their clinical management.

## Case presentation

A couple first attended our clinic in March 2023 seeking guidance regarding multiple failed attempts at conception and an ongoing pregnancy. The couple was of reproductive age (wife: 30 years; husband: 29 years) and reported two and a half years of active attempts at spontaneous conception, which had resulted in two miscarriages. There were no notable medical conditions in either partner’s history; neither was a smoker, and both reported maintaining a relatively healthy lifestyle.

As this was their first time undergoing fertility investigations and due to the wide range of potential underlying etiologic factors, a comprehensive diagnostic workup was initiated to guide clinical management. Initial gynecological examination revealed no abnormalities, and transvaginal 3D ultrasound demonstrated a normal endometrial cavity with no evidence of polyps or leiomyomas. A vaginal fluid culture identified *Ureaplasma urealyticum*, which was treated with a combination regimen of doxycycline and azithromycin for both partners.

Genetic testing, including molecular karyotyping (microarray analysis) and cystic fibrosis screening, was performed on both partners to assess carrier status. The results were normal: molecular karyotypes showed arr(1-22)x2,XX for the female and arr(1-22)x2,XY for the male; cystic fibrosis screening indicated ΔF508 normal for the female and 90% of CF mutations were ruled out in the male. Hemoglobin electrophoresis, performed to rule out hemoglobinopathies prevalent in the region, also returned normal results for both partners. In addition, semen analysis, sperm culture, and sperm DNA fragmentation index testing were performed on the husband, all of which were within normal parameters, excluding male factor infertility.

In the absence of any evident causes, thrombophilia testing was conducted one month later, revealing homozygosity for the C677T mutation of the *MTHFR *enzyme, along with elevated lipoprotein(a) levels (32.9). Repeat sperm and vaginal cultures were performed; the sperm culture was negative, while the vaginal culture tested positive for *Haemophilus vaginalis*. The woman was treated with clindamycin cream and metronidazole, and the couple was advised to attempt timed intercourse in the following cycle after completing antibiotic treatment.

After unsuccessful conception attempts, IVF treatment was recommended in June 2023. However, at the couple’s request, spontaneous conception efforts with timed intercourse were continued for several additional cycles. To further investigate the uterine environment, endometrial microbiome analysis using next-generation sequencing (NGS) was conducted. The results indicated chronic endometritis, with 96% *Enterococcus *and 4% other bacteria. Accordingly, the woman received a one-month treatment regimen that included ciprofloxacin, a combination of ampicillin and sulbactam, and lactobacillus supplements. The couple continued their attempts at conception per their preference.

In the subsequent months, the couple experienced three more pregnancy losses - one biochemical and two clinical pregnancies, which progressed to the sixth and eighth weeks of gestation, respectively. Karyotype analysis of the abortuses from the latter two losses revealed trisomy 16 and trisomy 13. Given this apparent genetic component, IVF with PGT for aneuploidy (PGT-A) was strongly recommended, and the couple agreed to proceed.

A standard short antagonist ovarian stimulation protocol was employed, yielding seven Day 5 blastocysts. Trophectoderm biopsies were collected, and the embryos were cryopreserved. Genetic analysis using NGS was performed on the biopsied material, and one month later, five euploid embryos were identified. Three months later, a single euploid embryo was transferred during a natural preparation cycle. A few days post-transfer, pregnancy was confirmed via a positive beta-hCG result, and the presence of an intrauterine gestational sac was visualized on ultrasound a few weeks later.

At the time of writing, the pregnancy is progressing uneventfully and is currently at 11 weeks of gestation under continued monitoring by our obstetrics division.

## Discussion

The present report highlights the utility and value of PGT-A in ensuring optimal embryo quality and improving fertility outcomes in cases of RPL, particularly when maternal factors such as endometrial receptivity have already been addressed. RPL remains a particularly complex and challenging clinical entity in the context of ART, necessitating thorough and attentive diagnostic investigation due to its multifactorial etiology.

Evaluation of RPL

Karyotyping

Karyotyping of the abortus or products of conception provides critical insights into the underlying cause of miscarriage. A normal karyotype suggests that pregnancy loss likely resulted from maternal, particularly endometrial, factors, thereby directing diagnostic efforts toward maternal abnormalities [11]. Conversely, the identification of an abnormal karyotype or aneuploidy, such as in the present case, implies an embryonic origin of RPL and usually suffices to explain a nonviable pregnancy [11,12].

Gynecological Imaging

Transvaginal ultrasound and hysterosalpingography are key imaging techniques used to diagnose uterine anomalies such as fibroids, adenomyosis, or intrauterine adhesions. Sonohysterography may be employed to assess fallopian tube patency and function [13]. Although hysteroscopy, laparoscopy, and MRI can also be used, they are more expensive, and the former two are more invasive. Ultrasonography remains the preferred option for pregnant patients, for whom other diagnostic methods are often contraindicated [14-16].

Immunological Testing

Testing for antiphospholipid syndrome involves measuring anti-beta2 glycoprotein I antibodies, anticardiolipin antibodies (IgG and IgM), and lupus anticoagulant. These tests should be repeated after 12 weeks, as transient low- to mid-positive levels may occur due to viral illnesses [17].

Hormonal Testing

Thyroid function should be evaluated in women with clinical symptoms or a personal history of thyroid disease. In asymptomatic women, screening is recommended to exclude subclinical thyroid dysfunction, which may be indicated by the presence of thyroid peroxidase autoantibodies [18].

Testing for Less Common Causes

Inherited thrombophilia should be evaluated in cases of recurrent, unexplained late fetal loss (beyond nine weeks of gestation), particularly when placental ischemia, infarction, or maternal vessel thrombosis is suspected. Anticoagulation treatment, initiated immediately after conception, is typically recommended for women with confirmed thrombophilia [19]. Screening for diabetes mellitus may be considered for women with suggestive clinical signs, as poorly controlled diabetes is known to increase miscarriage risk [20]. Although the male contribution to RPL remains unclear, sperm DNA fragmentation has been associated with miscarriage in some studies, which justified its evaluation in the present case [21,22].

PGT-A

PGT-A aims to detect de novo aneuploidies and subchromosomal deletions or duplications in embryos from phenotypically and karyotypically normal parents. By avoiding the transfer of aneuploid embryos, PGT-A seeks to reduce the risk of miscarriage and increase the likelihood of achieving a successful pregnancy.

In contemporary ART practice, PGT-A is typically performed at the blastocyst stage, five to six days post-fertilization. Trophectoderm cells are biopsied using gentle suction or mechanical compression, with five to eight cells usually extracted to minimize disruption to the developing fetoplacental unit. Compared to earlier-stage biopsies, blastocyst biopsy yields sufficient DNA for analysis, thus reducing diagnostic errors and being the least disruptive method for the embryo [23]. Following biopsy, embryos are cryopreserved while awaiting genetic analysis. If euploidy is confirmed, the embryo is then transferred. In some cases, previously cryopreserved embryos from prior stimulation cycles may be thawed, biopsied for PGT-A, cryopreserved again, and transferred later if euploid. Notably, this process does not significantly impact pregnancy or live birth rates compared to cases involving biopsy prior to the initial cryopreservation [24]. The biopsied cells are typically analyzed using NGS, which is considered the modern gold standard for genetic analysis [25].

As a diagnostic tool, PGT-A does not correct embryo ploidy and may not improve cumulative pregnancy or live birth rates. However, in patients with RPL, PGT-A has been shown to significantly reduce the miscarriage rate per embryo transfer (OR 0.42; 95% CI: 0.27-0.67) and improve live birth rates both per transfer (OR 2.17; 95% CI: 1.77-2.65) and per patient (OR 1.85; 95% CI: 1.18-2.91) [26], which aligns with the outcomes observed in the present case. Moreover, PGT-A allows for more confident adoption of single embryo transfer protocols, since euploid embryos have higher implantation potential than untested embryos, thereby reducing the number of embryos transferred and minimizing the risk of multiple gestations [27]. Because euploid embryos are less likely to result in miscarriage and more likely to lead to live birth, PGT-A also reduces the overall time to live birth [28] - a critical factor in ART, where time is often a pressing concern. Although time was not a primary issue in our case, the success of PGT-A in achieving an ongoing pregnancy, after multiple failed attempts, underscored its value in improving outcomes and preserving options for future attempts.

Nevertheless, like any diagnostic technique, PGT-A is not infallible and may result in misdiagnosis, although the actual incidence is difficult to quantify, as falsely identified aneuploid embryos are typically discarded [29,30]. While only euploid embryos are usually selected for transfer, mosaic embryos may be considered under special circumstances. Mosaicism, caused by mitotic errors after fertilization, results in embryos with a mix of euploid and aneuploid cell populations. Current NGS techniques can detect and quantify low-level mosaicism, which may be corrected naturally by embryonic mechanisms and could still lead to viable pregnancies [31]. In such cases, thorough patient counseling is essential, and any decision to transfer a mosaic embryo should be made jointly with the patient after discussion of all risks and limitations of the PGT-A technique [32].

## Conclusions

RPL presents a particularly complex challenge in ART due to its multifactorial nature. Comprehensive diagnostic testing is essential to investigate both maternal and fetal causes. As demonstrated in the present case, PGT-A serves as an effective embryo selection strategy when RPL is primarily attributable to fetal genetic factors. By enabling the transfer of euploid embryos, PGT-A has been shown to improve live birth rates per transfer and reduce the time to live birth.
